# Dynamic Contrast-Enhanced MRI Perfusion Parameters as Imaging Biomarkers of Angiogenesis

**DOI:** 10.1371/journal.pone.0168632

**Published:** 2016-12-30

**Authors:** Sung Hun Kim, Hyeon Sil Lee, Bong Joo Kang, Byung Joo Song, Hyun-Bin Kim, Hyunyong Lee, Min-Sun Jin, Ahwon Lee

**Affiliations:** 1 Department of Radiology, Seoul St. Mary’s Hospital, College of Medicine, The Catholic University of Korea, Seoul, Republic of Korea; 2 Deparment of General Surgery, Seoul St. Mary’s Hospital, College of Medicine, The Catholic University of Korea, Seoul, Republic of Korea; 3 Department of Biostatistics, Clinical Research Coordinating Center, College of Medicine, The Catholic University of Korea, Seoul, Republic of Korea; 4 Department of Hospital Pathology, Seoul St. Mary’s Hospital, College of Medicine, The Catholic University of Korea, Seoul, Republic of Korea; Kyoto Daigaku, JAPAN

## Abstract

Hypoxia in the tumor microenvironment is the leading factor in angiogenesis. Angiogenesis can be identified by dynamic contrast-enhanced breast MRI (DCE MRI). Here we investigate the relationship between perfusion parameters on DCE MRI and angiogenic and prognostic factors in patients with invasive ductal carcinoma (IDC). Perfusion parameters (K^trans^, k_ep_ and v_e_) of 81 IDC were obtained using histogram analysis. Twenty-fifth, 50th and 75th percentile values were calculated and were analyzed for association with microvessel density (MVD), vascular endothelial growth factor (VEGF) and conventional prognostic factors. Correlation between MVD and v_e50_ was positive (r = 0.33). K^trans^_50_ was higher in tumors larger than 2 cm than in tumors smaller than 2 cm. In multivariate analysis, K^trans^_50_ was affected by tumor size and MVD with 12.8% explanation. There was significant association between K^trans^_50_ and tumor size and MVD. Therefore we conclude that DCE MRI perfusion parameters are potential imaging biomarkers for prediction of tumor angiogenesis and aggressiveness.

## Introduction

Hypoxia in the tumor microenvironment exists due to structurally and functionally abnormal vessels, as well as oxygen consumption caused by rapid proliferation of tumor cells. By regulating the process of invasion and metastasis, hypoxia is the leading factor in angiogenesis. Vascular endothelial growth factor (VEGF) seems to be critical for blood vessel development, stimulating the formation of new blood and increasing vascular permeability[1–3]. Neoangiogenesis can be quantified using microvessel density (MVD)[3].

Angiogenesis can be identified by dynamic contrast-enhanced breast MRI (DCE MRI)[4]. Conventional contrast enhanced MRI analysis is based on subjective evaluation of signal enhancement curves, and is characterized by high spatial and low temporal resolution of 90–120 sec[5]. Although this approach is the most straightforward, it provides no quantifiable measurements[4]. Quantitative analyses can provide pharmacokinetic parameters that directly reflect the physiological properties of tissues, including vessel permeability, perfusion and the volume of extravascular/extracellular space (EES)[4].

Several studies have attempted to demonstrate correlation between DCE MRI perfusion parameters and angiogenic factors[6–10], with variable results. The only study regarding breast DCE MRI demonstrated positive correlation between K^trans^, k_ep_ and MVD in benign and malignant profiles of breast disease[7]. Previous studies placed a region of interest (ROI) in a small area surrounding tumor periphery or on the largest area on a single axial scan[8–10] and small areas of high K^trans^ values within the tumor[6]. These methods resulted in observer bias and insufficient information regarding tumor heterogeneity. To overcome these, we used histogram analysis of the entire tumor volume[11,12].

The goal of our study is to investigate the relationship between DCEMRI perfusion parameters and angiogenic factors (MVD, VEGF) and conventional prognostic factors in patients with invasive ductal carcinoma (IDC).

## Materials and Methods

### Patients

The study protocol was approved by the Institutional Review Board of Seoul St. Mary’s Hospital, and written informed consent was obtained from all patients. Between 2014 and 2015, 79 consecutive patients were considered for enrollment in our study: 1) IDC, not otherwise specified, was pathologically confirmed by means of percutaneous ultrasound guided biopsy; 2) maximum diameter between 1 and 5 cm; and 3) surgery was scheduled without neoadjuvant chemotherapy after MRI acquisition. Among the 79 eligible patients, we excluded six for the following reasons: failure of acquisition of perfusion parameters due to data-processing errors, systemic therapy with distant metastasis, and neoadjuvant chemotherapy. A total of 73 patients with 81 total lesions were included in our study: two cancers in 4 patients, three cancers in one patient and multifocal malignancy and bilateral cancer in one patient.

### MR Image Acquisition

MR examinations were performed in the prone position using a 3T system (Magnetom Verio; Siemens Healthcare, Erlangen, Germany) and a dedicated eight-channel phase-array coil. The images were obtained using the following sequences: (1) axial turbo spin-echo T2-weighted imaging (T2WI) sequence with TR/TE of 4530/93 msec, flip angle of 80°, FOV of 320 x 320 mm^2^, matrix size of 576 × 403, slice thickness of 4mm, and acquisition time of 2 min 28 sec; (2) pre-contrast T1-weighted 3D volumetric interpolated breath-hold examinations (3D VIBE) with TR/TE of 2.7/0.8 msec, FOV of 320 x 320 mm^2^, matrix size of 256 x 192, slice thickness of 2 mm with various flip angles (2°, 6°, 9°, 12°, 15°), and acquisition time of 2 min 15 sec to determine tissue T1 relaxation time prior to the arrival of contrast agent; (3) dynamic contrast-enhanced axial T1-weighted imaging (T1WI) with fat suppression with TR/TE of 2.5/0.8 msec, flip angle of 10°, slice thickness of 2.0 mm, and acquisition time of 5 min 30 sec (temporal resolution 6 sec) following an intravenous bolus injection of 0.1 mmol/kg gadobutol (Gadovist, Schering, Berlin, Germany) followed by a 20 ml saline flush; (4) delayed axialT1-weighted 3D VIBE sequence with TR/TE of 4.4/1.7msec, flip angle of 10°, slice thickness of 1.2 mm, field of view of 340 mm, and matrix size of448 x 358 to evaluate the overall extent of tumor.

### Image Analysis

**Fig 1:**
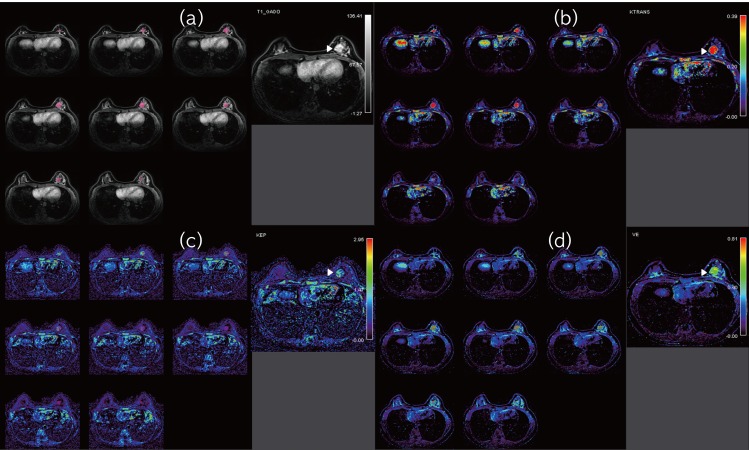
Example of perfusion parameter calculation using the software. (a). Early contrast enhanced T1 WI with fat suppression shows left breast cancer (arrowhead). A volume of interest (VOI) covering whole tumor area was semiautomatically drawn on the early contrast enhancement phase images in pink. (b-d). The VOI was copied to the corresponding Ktrans–, kep–, and ve–based perfusion map.

MRI data were evaluated by two radiologists (SHK, HSL) in consensus, with 10 years and one year of experience with breast MRIs, respectively. The radiologists were blinded to clinical information including molecular markers and angiogenic factors. Perfusion parameters were quantitatively analyzed using dedicated DCEMRI software (Olea Sphere 2.3, Olea Medical SAS, La Ciotat, France), based on the extended Tofts mathematical model. Native T1 maps were generated using the five flip-angles. The arterial input function (AIF) was obtained from the aorta or axillary artery using an automatic AIF selection algorithm implemented in the software. Three perfusion parameters were used to assess tissue and vascular permeability characteristics: (1) K^trans^ (min^-1^, volume transfer constant from blood plasma to EES); (2) k_ep_ (min^-1^, rate constant from EES to plasma); (3) v_e_ (ml/100ml of tissue; %, volume of EES per unit volume of tissue)[13,14]. We drew a volume of interest (VOI) encompassing the entire tumor volume on the early contrast enhancement phase (90 sec following contrast injection), and the VOI was copied to the corresponding K^trans^–, k_ep_–, and v_e_–based perfusion maps (Fig 1). The perfusion parameter value of each voxel and a histogram of the perfusion parameter data were generated for the entire tumor volume. Histogram analysis was performed and twenty-fifth percentile, 50th percentile and 75th percentile values were calculated as the cumulative parameters.

### Pathologic Analysis

Histopathological assessment of surgical specimens was performed by a pathologist (AL) with 15 years of experience. Estrogen (ER) and progesterone (PR) positivity were defined as stained nuclei in more than 1% of invasive cancer cells on an entire stained slide. The intensity of HER2 expression was semi-quantitatively scored as 0, 1+, 2+, or 3+. Cancers with a 3+ score were classified as HER2 positive, and those with a score of 0 or 1+ were considered HER2 negative. Gene amplification using dual-color silver *in situ* hybridization (SISH) with an automated Ventana INFORM HER2 Genomic probe platform (Tucson, Arizona, USA) was performed to determine HER2 status in cancers with a score of 2+. HER2 expression was considered positive if the signal ratio of HER2 genes copied to chromosome 17 was greater than two. Tumor subtypes were categorized by molecular marker expression as follows: luminal type, HER2 enriched type and triple-negative type. Immunohistochemical staining of CD34 and VEGF was performed. The antibodies and dilutions used were: CD34 (clone QBEnd 10, 1:100, DAKO, Carpinteria, CA, USA) and VEGF (clone A-20, 1:200; Santa Cruz, Heidelberg, Germany). VEGF expression levels were determined semi-quantitatively by adding the fraction and intensity scores, which is the modified scoring system of Klein et al[15]. The fraction score was determined by the positive staining fraction of tumor cells (score 0 = no staining, score 1 = 1–10%, score 2 = 11–33%, score 3 = 34–66%, score 4 = 67–100%). The intensity score was determined by the staining intensity of tumor cells (score 0 = no staining, score 1 = weak staining, score 2 = moderate staining, score 3 = strong staining). MVD was determined from the CD34 immunohistochemical-staining slides. A single countable vessel was defined as any positively stained endothelial cell or cell cluster separate from adjacent microvessels or tumor cells. The vessels containing erythrocytes in the lumen were excluded[16]. Five high power fields were counted, and the average was determined.

### Statistical Analysis

MVD and VEGF staining score were considered continuous variables.

Cases were assigned to one of two groups according to the dichotomized histopathologic prognostic factors and subtypes: tumor size (≤2cm vs.>2cm), axillary node status (negative vs. positive), histologic grade (grades 1 and 2 vs. grade 3), ER, PR, and HER-2 expression (negative vs. positive). Subtypes were analyzed by paired comparison: luminal (ER and/or PR positive), HER2 enriched (HER2 over-expressed or amplified, ER and PR absent) and triple-negative type(ER and PR absent, HER2 negative).

The normality of continuous variables (perfusion parameters) was verified, and the variables were transformed (e.g., log transformed) if necessary. Descriptive characteristics are presented as the mean ± standard deviation (SD) for parameteric variables or median and interquartile range for non-parametric variables based on normality (Shapiro-Wilk test). Pearson’s or Spearman’s correlation coefficient was calculated to determine the relationship between perfusion parameters and angiogenic factors. The significant threshold for correlation was considered at a r-value more than 0.25. To compare the prognostic factor and perfusion parameters, group differences test was performed (e.g.; Student’s t-test, Wilcoxon rank sum test for two group, ANOVA, and Kruskal-Wallis test for three group), followed by post-hoc analysis with Bonferroni correction in case of three group comparison. The significant threshold for difference was set at a p-value less than 0.0056 (0.05/9) for multiple comparison correction of nine perfusion parameters. Perfusion parameter with skewed distributions was simple and multiple linear regression models after log-transformation of the data were used. A multiple linear regression model was constructed, using statistically significant variables from univariate analysis. All statistical analyses were performed using the software package SAS Enterprise Guide 5.1 (SAS Institute, Inc, Cary, NC).

## Results

### Patients

The mean patient age was 53.3 ± 10.0 years (range, 34–77 years). Mean tumor size was 2.2 ± 0.9 cm (range, 1–5 cm), and 56 (69.1%) IDCs exhibited a DCIS component.

### Perfusion Parameters and Angiogenic Factors

**Fig 2:**
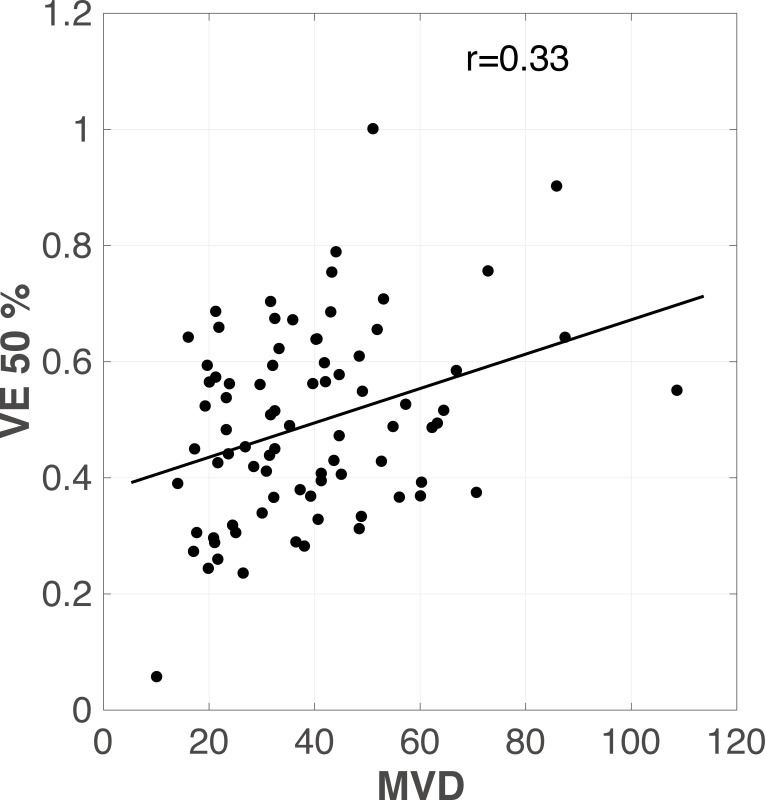
Scatterplot of v_e_ vs. angiogenic factor. Correlation between microvessel density (MVD) and v_e50_ is positive (r = 0.33).

Correlation between MVD and v_e50_ was positive (r = 0.33) (Fig 2). Correlation between MVD and K^trans^_50,_ andv_e75_ was weakly positive (r = 0.25 and 0.26, respectively). There was no significant correlation between MVD or VEGF and other perfusion parameters (Table 1).

**Table 1:** Correlation between perfusion parameters and angiogenesis factors.

Variables	n	Ktrans			kep			ve		
		25%	50%	75%	25%	50%	75%	25%	50%	75%
MVD	81	0.19 (0.085)	**0.25** (0.022)	0.24 (0.024)	0.08 (0.431)	0.10 (0.352)	0.17 (0.129)	0.20 (0.071)	**0.33** (0.002)	**0.26** (0.015)
VEGF	81	-0.04 (0.713)	-0.029 (0.7907)	-0.05 (0.617)	0.18 (0.105)	0.24 (0.025)	0.22 (0.040)	-0.24 (0.027)	-0.20 (0.066)	-0.19 (0.083)

Note—Data are presented as r (p-value).

The statistical tests were carried out using Pearson's or Spearman's correlation analysis. The significance threshold for correlation was set at a r-value more than 0.25.

### Perfusion Parameters and Conventional Prognostic Factors

K^trans^_50_ was higher in tumors larger than 2 cm (0.26, range 0.17–0.41) than in tumors smaller than 2 cm (0.18, range 0.13–0.25; p = 0.004).

Other prognostic factors were not significantly associated with various K^trans^ values.

There was no significant association among various k_ep,_ v_e_ values and prognostic factors (Tables 2 and 3).

**Table 2:** Correlation between various perfusion parameters and conventional prognositc factors.

Variables		No of cases(%)	Median(range)							Mean (±SD)	
			Ktrans 25 percentile	Ktrans 50 percentile	Ktrans 75 percentile	Kep 25 percentile	Kep 50 percentile	Kep 75 percentile	ve 25 percentile	ve 50 percentile	ve 75 percentile
Tumor size(mm)	≤20	36 (44.4)	0.10 (0.08–0.15)	0.18 (0.13–0.25)	0.30 (0.21–0.42)	0.26 (0.21–0.36)	0.46 (0.32–0.57)	0.67 (0.48–0.78)	0.29 (0.23–0.40)	0.45±0.15	0.58±0.16
	>20	45 (55.6)	0.12 (0.10–0.19)	0.26 (0.17–0.41)	0.36 (0.24–0.55)	0.30 (0.24–0.39)	0.48 (0.39–0.69)	0.77 (0.58–1.11)	0.36 (0.30–0.44)	0.52±0.16	0.63±0.18
*p*-value			0.035	**0.004**	0.063	0.116	0.066	0.062	0.058	0.042	0.143
Lymph node metastasis	Negative	46 (56.8)	0.13 (0.08–0.19)	0.24 (0.17–0.36)	0.37 (0.23–0.49)	0.30 (0.23–0.42)	0.46 (0.37–0.66)	0.68 (0.57–0.91)	0.32 (0.25–0.41)	0.49±0.17	0.61±0.17
	Positive	35 (43.2)	0.11 (0.08–0.15)	0.18 (0.15–0.31)	0.30 (0.21–0.42)	0.26 (0.23–0.35)	0.47 (0.36–0.61)	0.69 (0.48–0.90)	0.38 (0.25–0.44)	0.49±0.16	0.61±0.18
*p*-value			0.180	0.210	0.217	0.279	0.564	0.860	0.415	0.952	0.965
Histologic grade	1 or 2	45 (55.6)	0.11 (0.08–0.19)	0.20 (0.15–0.33)	0.32 (0.23–0.46)	0.28 (0.23–0.35)	0.46 (0.36–0.59)	0.64 (0.48–0.87)	0.35 (0.27–0.42)	0.50±0.17	0.62±0.16
	3	36 (44.4)	0.12 (0.09–0.17)	0.22 (0.15–0.33)	0.35 (0.23–0.46)	0.31 (0.24–0.39)	0.50 (0.41–0.63)	0.77 (0.62–0.94)	0.32 (0.25–0.43)	0.48±0.15	0.60±0.19
*p*-value			0.433	0.794	0.981	0.237	0.215	0.132	0.618	0.500	0.470

Note—Data are presented as median (interquartile range) or mean±SD. Numbers in parenthesis are percentage.

The statistical tests were carried out using Student t test or Wilcoxon rank sum test for two group comparison. The significance threshold for difference was set at a p-value less than 0.0056 (0.05/9) for multiple comparison correction of nine perfusion parameters.

**Table 3:** Correlation between various perfusion parameters and molecular markers and subtypes.

Variables		No of cases(%)	Median(range)							Mean (±SD)	
			Ktrans 25 percentile	Ktrans 50 percentile	Ktrans 75 percentile	Kep 25 percentile	Kep 50 percentile	Kep 75 percentile	ve 25 percentile	ve 50 percentile	ve 75 percentile
ER	Negative	20 (24.7)	0.10 (0.06–0.15)	0.17 (0.11–0.28)	0.26 (0.17–0.44)	0.24 (0.18–0.30)	0.44 (0.31–0.48)	0.65 (0.39–0.81)	0.30 (0.17–0.39)	0.43±0.16	0.58±0.16
	Positive	61 (75.3)	0.12 (0.08–0.19)	0.23 (0.17–0.34)	0.33 (0.25–0.48)	0.31 (0.23–0.40)	0.48 (0.39–0.66)	0.71 (0.57–0.97)	0.35 (0.27–0.44)	0.51±0.16	0.62±0.18
*p*-value			0.083	0.030	0.085	0.017	0.036	0.218	0.065	0.062	0.393
PR	Negative	28 (34.6)	0.10 (0.08–0.16)	0.19 (0.13–0.30)	0.31 (0.20–0.44)	0.29 (0.22–0.34)	0.47 (0.36–0.59)	0.71 (0.52–0.89)	0.30 (0.23–0.39)	0.45±0.18	0.57±0.18
	Positive	53 (65.4)	0.12 (0.08–0.19)	0.23 (0.15–0.36)	0.33 (0.24–0.48)	0.30 (0.23–0.40)	0.47 (0.37–0.68)	0.67 (0.56–0.97)	0.35 (0.27–0.44)	0.51±0.15	0.63±0.17
*p*-value			0.418	0.157	0.195	0.343	0.453	0.777	0.127	0.082	0.155
HER2	Negative	59 (72.8)	0.11 (0.08–0.19)	0.20 (0.15–0.34)	0.32 (0.22–0.48)	0.29 (0.23–0.38)	0.46 (0.36–0.6)	0.66 (0.49–0.97)	0.34 (0.25–0.41)	0.49±0.17	0.61±0.17
	Positive	22 (27.2)	0.11 (0.09–0.16)	0.23 (0.15–0.31)	0.36 (0.24–0.46)	0.29 (0.24–0.37)	0.48 (0.44–0.60)	0.77 (0.64–0.89)	0.36 (0.26–0.44)	0.50±0.15	0.61±0.18
*p*-value			0.979	0.937	0.996	0.738	0.614	0.521	0.563	0.858	0.982
Subtype	Luminal	61 (75.3)	0.12 (0.08–0.19)	0.23 (0.17–0.34)	0.33 (0.25–0.48)	0.31 (0.23–0.40)	0.48 (0.39–0.66)	0.71 (0.44–0.88)	0.35 (0.27–0.44)	0.51±0.16	0.62±0.18
	Triple negative	11 (13.6)	0.09 (0.04–0.15)	0.15 (0.08–0.30)	0.21 (0.15–0.44)	0.22 (0.10–0.33)	0.38 (0.20–0.56)	0.64 (0.36–0.90)	0.25 (0.16–0.35)	0.39±0.17	0.56±0.17
	HER2 enriched	9 (11.1)	0.10 (0.09–0.16)	0.17 (0.13–0.26)	0.27 (0.23–0.37)	0.28 (0.24–0.29)	0.45 (0.43–0.48)	0.66 (0.64–0.79)	0.34 (0.28–0.41)	0.48±0.13	0.61±0.15
*p*-value*			0.197	0.092	0.215	0.048	0.106	0.457	0.050	0.081	0.570

Note—Note—Data are presented as median (interquartile range) or mean±SD. Numbers in parenthesis are percentage.

The statistical tests were carried out using Student t test or Wilcoxon rank sum test for two group, ANOVA test or Kruskal-Wallis test for three group comparison. The significance threshold for difference was set at a p-value less than 0.0056 (0.05/9) for multiple comparison correction of nine perfusion parameters.

*P-value of 0.0018 was considered to indicate statistical significance accounting for a Bonferroni correction

ER, estrogen receptor; PR, progesterone receptor; HER2, human epidermal growth factor receptor 2

### Multivariate Regression Analysis

**Fig 3:**
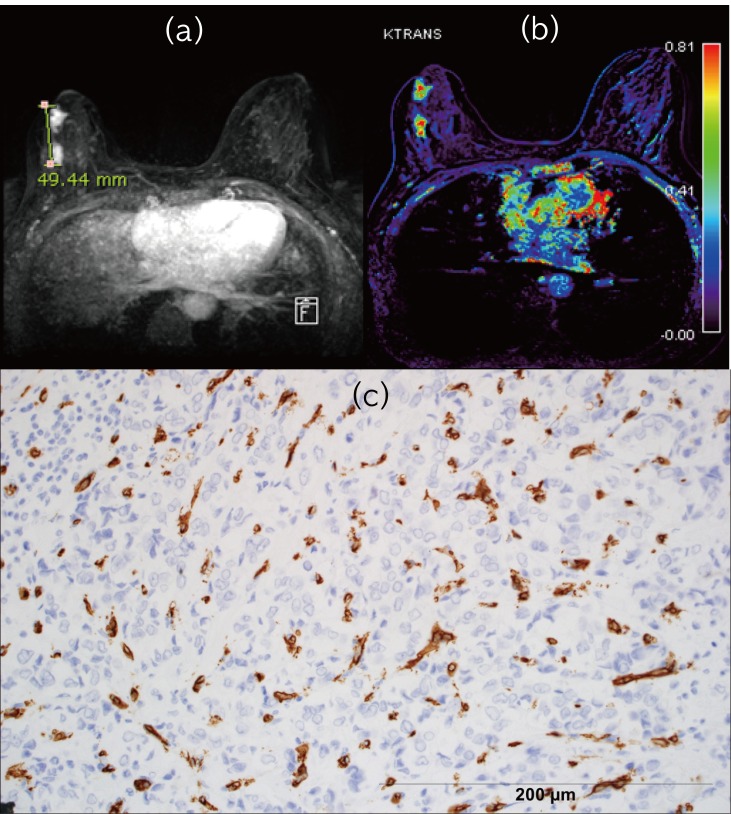
A 44 year-old woman with large tumor size, high K^trans^ and microvessel density (MVD). (a). Maximal intensity projection image of early contrast phase enhancement phase shows a 4.9 cm sized invasive ductal carcinoma in right breast. Two neighboring masses are connected on pathology. (b). K^trans^ map demonstrates red color on the tumor (K^trans^_50_ = 0.568) (c). Photomicrograph with CD34 shows vascular endothelium staining as dark brown (microvessel density 86) (Original magnification X200).

Multiple linear regression analysis was performed. Prognostic factors that demonstrated significant differences in univariate analysis were included (Table 4). Tumor size and MVD were significantly associated with elevated K^trans^_50_ values (p<0.05) (Fig 3). K^trans^_50_ was affected by tumor size and MVD with 12.8% explanation.

**Table 4:** Multiple linear regression analysis of perfusion parameters and angiogenesis, prognostic factors.

Variable	Ktrans 50 percentile		
	B	SE	*p*-value
Tumor size (>2cm)	0.365	0.131	**0.007**
MVD	0.008	0.004	**0.026**
AdjR2(%)	12.8		

Note–MVD, microvessel density; B, Beta coefficient; SE, standardized error; Adj R, adjusted coefficient of determination

## Discussion

Our study was designed to demonstrate the relationship between DCE-MRI perfusion parameters and angiogenic factors. K^trans^_50_ value in IDCs demonstrated a meaningful positive correlation with MVD. K^trans^ is known to be influenced by blood flow, vessel surface area and vessel permeability and has been selected as the preferred DCE-MRI end points in clinical trials as biomarker of tumor perfusion and permeability[17,18]. MVD is considered to quantify the tumor microvasculature and to permit estimation of tumor angiogenesis[7]. This result was consistent with the previous study[7]. This differed from other studies: negative correlation between K^trans^ and MVD[9] and no association [6,8,19] (Table5).

**Table 5:** Correlative studies between perfusion parameters and angiogenesis factors.

	George's [10]	Atkin's (9)	Yeo's [6]	Oto's [19]	Haldorsen's [8]	Li's [7]	Present study
Organ	Retal Cancer	Rectal Cancer	Rectal Cancer	Prostate Cancer, Benign tissue	Endometial Cancer	Breast Cancer, Benign Disease	Breast Cancer
Case number	31	12	32	73	54	59(cancer), 65(benign)	81
MRI	1.5 T	1.5 T	3T	1.5 T	1.5 T	3T	3T
DCE model	Tofts	Tofts	Tofts	Tofts	Johnson and Wilson	NM	Tofts
Perfusion parameters	Ktran	Ktrans, ve	Ktrans, kep, ve, iAUC	Ktrans, kep,ve	Ktrans, kep, ve, iAUC	Ktrans, kep, ve	Ktrans, kep, ve
ROI	tumor periphery	tumor periphery	entire tumor on section	largest area	small area	NM	tumor volume
Angiogenesis factors	VEGF(serum)	MVD(CD31) VEGF	MVD(CD31) VEGF	MVD(CD31,CD34)VEGF	MVD(FVIII)	MVD(CD31) MVD(CD105)	MVD(CD34) VEGF
Results	P (Ktrans,VEGF)	N(Ktans,MVD) No-others	P(kep,MVD) No-others	P(kep,MVD) No-others	No-all	P(Ktrans,MVD)(CD105) P(kep,MVD)(CD105) No-others	P(ve,MVD) P(Ktans, MVD) No-others

Note–NM, not mentioned; ROI, region of interest on DCE MRI; MV, microvesseldensi; VEGF, vascular endothelial growth factor; P, positive correlation, negative correlate; No, no correlation

v_e50_ demonstrated a positive correlation with MVD (r = 0.33) in the present study. ve represents the volume of EES per unit volume of tissue[13,14,17]. Higher v_e_ values were thought to be associated with lower tumor cellularity and a rich stroma; the latter is composed of fibroblasts, endothelium and extracellular matrix components that supply the tumor with growth factors and stimulate the formation of blood vessels. This activation occurs in large areas of breast tissue[20], which may explain the positive association between v_e50_ and MVD. However, our results differed from previous results that showed no correlation between v_e_ and MVD[6–9,19] (Table 5).Tumor angiogenesis is known to arise via upregulation of VEGF and the expression level of VEGF has been shown to correlate with MVD[6,21]. In rectal cancer, positive correlation between K^trans^ and VEGF was reported[10]. However, our study demonstrated no association between MR perfusion parameters and VEGF and agreed with those of an earlier study[6,9] (Table 5).

With regard to conventional prognostic factors, we found elevated K^trans^_50_values in IDCs with tumors larger than 2 cm. Because angiogenesis is an essential process for tumor growth, these results are not surprising, considering that larger tumors would yield higher perfusion-parameter values on DCE-MRI. However, these results differed in previous studies, none of which demonstrated correlation with tumor size[13,22,23](Table 6).

**Table 6:** Correlative studies between perfusion parameters on breast DCE MRI and prognostic factors.

	Li's [27]	Koo's [23]	Kim's [13]	Yim's [22]	Present study
Inclusion	Invasive cancer	Invasive cancer	DCIS, Invasive cancer	Invasive cancer	Invasive ductal carcinoma
Case number	37	70	50	64	81
MRI	1.5T	1.5T	3T	1.5T	3T
DCE model	Tofts	Tofts	Tofts	NM	Tofts
Perfusion parameters	Ktrans, kep, ve, iAUC	Ktrans, kep, ve	Ktrans, kep, ve, iAUC	kep	Ktrans, kep, ve
ROI	entire tumor on section	largest area on section	small area with high Ktrans	tumor margin	tumor volume
Prognostic factors	subtype(TNC,L)	tumor size, LN, HG, NG, ER, PR, Ki-67, p53, Bcl-2, HER2, subtype (TNC,L,HER2)	tumor size, LN, HG, NG, ER, PR, HER2, EGFR, Bcl, CK5/6, Ki-67, subtype (TNC,L,HER2)	tumor size, HG, ER, HER2	tumor size, LN, HG, ER, PR, HER2, subtype (TNC,L,HER2)
Results	P(kep,TNC) N(ve,TNC) No-others	N(Ktrans,ER positivity) P(kep, HG) N(kep, ER positivity) P(kep, TNC)-TNC vs. L N(ve, TNC)-TNC vs. L	P(Ktrans, Ki-67 positivity) P(kep, ER positivity) P(kep, Ki-67 positivity) No-others	No-all	P(Ktrans, tumor size) No-others

Note–M, not mentioned; ROI, region of interest on DCE MRI;LN,lymph node; HG, histologic grade; NG, nucelar grade; TNC, triple negative cancer subtype; L, luminal type; HER2, Her2 enriched type; P, positive correlation; N, negative correlation; No, no correlation

ER has been reported to inhibit angiogenesis[24] and has been associated with high cellularity[25]. HER2 expression is known to increase angiogenesis[26]. We expected ER-positive tumors to be associated with low K^trans^, k_ep_ and v_e_ values and HER2-positive tumors to be associated with high K^trans^ and k_ep_ values. Lower v_e_ values in triple-negative type tumors describe the reduced extracellular space, and are consistent with a more compact cellularity. ve has been reported to be the best predictor of triple-negativity among perfusion parameters[27]. However, our study demonstrated no association among them.

Some of our results may be explained by the presumed roles of angiogenic and conventional factors, though some were contrary to our expectation. The unexpected results are likely due to the complex interaction between angiogenic factors, tumor cells and stromal cells. There were variable results in previous correlative studies, which may be attributed to differences in composition of study populations, as well as to differences in MR machines and parameters. Different ROIs were used for quantification. Additionally, there was no standard method for MVD quantification; in particular, antibody type and areas chosen for vessel count were highly variable[19].

There are some limitations to our study. First, we performed the volume-based histogram analysis while the representative portion of the tumor vascular profile was selected for MVD and VEGF measurements. Thus, angiogenic factors might not accurately reflect tumor heterogeneity. Second, we did not evaluate the interobserver variability to verify the reproducibility.

In conclusion, histogram analysis revealed meaningful association between perfusion parameter and angiogenic and prognostic factor. IDCs with elevated K^trans^_50_ were associated with higher MVD and larger tumor size. DCE MRI perfusion parameters have clinical potential as imaging biomarkers for prediction of tumor angiogenesis and aggressiveness.

## Supporting Information

S1 Dataset This is the basic dataset of this study including pathologic and radiologic information. (XLSX)
